# Comparative RNA-Seq Analysis Reveals That Regulatory Network of Maize Root Development Controls the Expression of Genes in Response to N Stress

**DOI:** 10.1371/journal.pone.0151697

**Published:** 2016-03-18

**Authors:** Xiujing He, Haixia Ma, Xiongwei Zhao, Shujun Nie, Yuhua Li, Zhiming Zhang, Yaou Shen, Qi Chen, Yanli Lu, Hai Lan, Shufeng Zhou, Shibin Gao, Guangtang Pan, Haijian Lin

**Affiliations:** Key Laboratory of Biology and Genetic Improvement of Maize in Southwest Region, Ministry of Agriculture; Maize Research Institute of Sichuan Agricultural University, Wenjiang, Sichuan, China; University of Missouri, UNITED STATES

## Abstract

Nitrogen (N) is an essential nutrient for plants, and it directly affects grain yield and protein content in cereal crops. Plant root systems are not only critical for anchorage in the soil, but also for N acquisition. Therefore, genes controlling root development might also affect N uptake by plants. In this study, the responses of nitrogen on root architecture of mutant *rtcs* and wild-type of maize were investigated by morphological and physiological analysis. Subsequently, we performed a comparative RNA-Seq analysis to compare gene expression profiles between mutant *rtcs* roots and wild-type roots under different N conditions. We identified 786 co-modulated differentially expressed genes (DEGs) related to root development. These genes participated in various metabolic processes. A co-expression cluster analysis and a cis-regulatory motifs analysis revealed the importance of the AP2-EREBP transcription factor family in the rtcs-dependent regulatory network. Some genotype-specific DEGs contained at least one LBD motif in their promoter region. Further analyses of the differences in gene transcript levels between *rtcs* and wild-type under different N conditions revealed 403 co-modulated DEGs with distinct functions. A comparative analysis revealed that the regulatory network controlling root development also controlled gene expression in response to N-deficiency. Several AP2-EREBP family members involved in multiple hormone signaling pathways were among the DEGs. These transcription factors might play important roles in the rtcs-dependent regulatory network related to root development and the N-deficiency response. Genes encoding the nitrate transporters NRT2-1, NAR2.1, NAR2.2, and NAR2.3 showed much higher transcript levels in *rtcs* than in wild-type under normal-N conditions. This result indicated that the LBD gene family mainly functions as transcriptional repressors, as noted in other studies. In summary, using a comparative RNA-Seq-based approach, we identified DEGs related to root development that also participated in the N-deficiency response in maize. These findings will increase our understanding of the molecular regulatory networks controlling root development and N-stress responses.

## Introduction

Nitrogen (N) is one of the most important nutrients for plants, and it directly affects grain yield and protein content in cereal crops [1]. Agricultural productivity is limited by N, as it is an essential macronutrient. Both low- and high-N supply can negatively affect plant growth. Deprivation of N can result in reduced protein and chlorophyll contents, and disruption of important biological processes such as N metabolism and photosynthesis [2, 3]. Previous studies have shown that excess N can negatively affect plant growth [4, 5]. Changes in the levels of plant hormones in response to excess N are thought to be one explanation for the negative effects [5, 6]. In addition, intensified use of N fertilizers in agriculture is already adversely affecting ecosystem diversity to some degree. To optimize the use of fertilizers and decrease their environmental impacts, it is very important to improve the N use efficiency (NUE) of plants. Understanding of the molecular mechanisms of N metabolism such as N uptake, assimilation, and recycling during plant growth and development will contribute to increasing the NUE of crop plants.

Various studies have focused on the physiological and molecular basis of nitrate uptake, allocation, and storage in higher plants. Four families of nitrate-transporting proteins have been identified: the nitrate transporter 1/peptide transporter (NRT1/PTR) family, the nitrate transporter 2 family (NRT2), the chloride channel (CLC) family, and the slow anion channel-associated homologs (SLAC1/SLAH) [7, 8]. The first nitrate transporter to be identified was chlorate resistant 1, which belongs to the NRT1/PTR family [7]. In addition to nitrate, NRT1/PTR proteins can transport a wide variety of substrates in plants, such as peptides, amino acids, dicarboxylates, glucosinolates, auxin (IAA), and abscisic acid (ABA) [9]. In contrast to NRT1/PTR transporters, the NRT2 transporters show strong substrate specificity. All Arabidopsis NRT2 transporters show high-affinity for nitrate, and no other substrate has been identified so far [8]. NAR2, a two-transmembrane-domain protein, is required for the nitrate transport activity of NRT2 transporters in higher plants [10, 11]. NAR2 is involved in targeting NRT2 to the plasma membrane, and/or in stabilizing NRT2 [7, 12].

Plant root growth and development are not only critical to anchor the plant in the soil, but also for N acquisition. Therefore, genes controlling root architecture and development may also affect nitrate uptake in plants. Identifying the genes involved in root development may be useful for the development of strategies to control NUE. The maize root system has a unique architecture that is necessary for effective uptake of water and nutrients to maximize biomass and yields. The maize root system consists of different types of roots: embryonic primary and seminal roots, post-embryonic shoot-borne roots, and lateral roots [13, 14]. These different types of roots have distinct functions during different stages of plant development. An ideotype root system for efficient acquisition of N and water in maize was proposed, abbreviated as SCD (steep, cheap, and deep), which integrates root architectural, anatomical, and physiological traits to enhance the rapid exploitation of deep soil strata [15]. Maize genotypes with few crown roots had greater rooting depth and nitrogen acquisition in low-N soils, resulting in greater photosynthesis and plant N content [16]. Root cortical aerenchyma improves maize growth under low-N conditions by decreasing root metabolic costs and increasing rooting depth, thereby enhancing soil exploration and N acquisition from low-N soils [17]. A number of maize mutants with defects in various aspects of root development were identified, which provided an excellent opportunity to investigate the impact of root morphological alterations on N acquisition and response of maize roots to N stress. The mutant *rtcs* (*rootless concerning crown and seminal roots*) lacks embryonic seminal and post-embryonic shoot-borne roots, and the *rtcs* gene was isolated from maize by map-based cloning [14, 18, 19]. The *rtcs* gene encodes a 244-amino-acid Lateral Organ Boundaries (LOB) domain (LBD) transcription factor that is involved in the initiation and maintenance of seminal and shoot-borne roots [19]. Members of the plant-specific LBD gene family are characterized by the LOB domain, which has a C-motif that is probably responsible for DNA binding, a conserved glycine residue, and a putative leucine zipper-like oligomerization domain [20, 21]. It has been demonstrated that LBD proteins could recognize a specific 5′-GCGGCG-3′ sequence (LBD motif) in the promoter region of target genes [22]. The special structure and nuclear localization of LBD proteins suggest that they are transcription factors, which probably function early in auxin signaling in different developmental stages of the root [23, 24]. The research on N stress responses in mutant *rtcs* is useful in further clarifying the interaction mechanism of root development and N acquisition.

RNA-Seq, as the technology of choice for whole-transcriptome studies, has been successfully applied in root development and N stress responses research, providing accurate analysis of gene expression profiling [25, 26]. To investigate the difference of root morphological and transcriptomic responses to low-N stress between the maize mutant *rtcs* and its wild-type, we explored the changes of root morphology and identified genotype- and/or treatment-related differentially expressed genes (DEGs). We further analyzed differences in gene regulation by constructing gene co-expression networks and by a functional enrichment analysis, which allowed us to infer the functions of genes related to root development and the N-deficiency response. Further research focused on the role of the *rtcs*-dependent regulatory network in regulating maize response to low-N stress. Comparative transcriptome analysis revealed that 245 co-modulated DEGs associated with root development participate in the response to N-deficiency. The information obtained in this study provides details of the mechanism of the N-deficiency response in plant roots. The genes identified in this study will be useful candidates for further studies on the molecular basis of root initiation in maize.

## Materials and Methods

### Plant materials preparation

The seeds of maize mutant *rtcs* and wild-type (inbred line B73) were used in these experiments. Seeds of mutant *rtcs* were obtained from the Maize Genetics Cooperation Stock Center (http://www.uiuc.edu/ph/www/maize/). Seeds were surface-sterilized in 10% (v/v) H_2_O_2_ for 40 min, rinsed five times by distilled water, and germinated at 28°C with a 14/10 h light/dark photoperiod. When two leaves were visible, the uniform seedlings were removed from endosperm and transferred into porcelain pots containing 2 L of nutrient solution. Seedlings with uniform growth were picked randomly and divided into the normal nitrogen group (N-4mM, normal-N) and the low nitrogen group (N-0.04mM, low-N). Ca(NO_3_)_2_·4H_2_O was used as nitrogen source, and the Ca^2-^ deficiency was supplemented with CaCl_2_ (see S1 Table). These plants were grown in at 28/22°C with a 14/10 h light/dark cycle and 70% humidity treatment. The nutrient solution was resupplied every other day and was aerated continuously.

### Morphological and physiological studies

Roots of wild-type and *rtcs* were harvested at 12 h, 24 h, 48 h and 96 h after two N treatments. The length of primary root (PRL) and total lateral root on primary root (LRL) were measured with the root scanner (EPSON EXPRESSION) and WinRHIZO Pro 5.0 (Quebec City, Canada). Three replicate pots with three seedlings per pot were selected randomly and sampled. The total protein content and glutamine synthetase (GS) activity were selected as representative indicators of primary nitrogen assimilation in maize roots. There were three replicates with a random design, and each biological replicate constituted a pool of three seedlings. The total protein content and GS activity were measured by total protein assay kit (Nanjing Jiancheng, China) and glutamine synthetase assay kit (Nanjing Jiancheng, China), respectively, following the manufacturer’s protocol. Statistical analyses were performed using SAS statistical software.

### RNA-Seq library construction and transcriptome sequencing

For the RNA-Seq, the roots of mutant *rtcs* and wild-type in normal-N and low-N group were sampled and immediately frozen with liquid nitrogen at 12 h, 24 h, 48 h and 96 h after two N treatments, and labelled as RNI-IV, RLI-IV, WNI-IV and WLI-IV, successively. All samples were harvested from each of the three maize seedlings, and two independent replicates were collected for each sample. Total RNA of the roots was isolated with Trizol reagent (Invitrogen, USA) according to the manufacturer’s protocol. The transcriptome sequencing library was generated using NEBNext Ultra RNA Library Prep Kits for Illumina (NEB, USA). In brief, polyadenylated RNA purification, RNA fragmentation, cDNA synthesis, and polymerase chain reaction (PCR) amplification were performed according to the Illumina RNA-Seq protocol. 125bp paired-end reads were generated using Illumina HiSeq 2500 (Novogene, China).

### Reads preprocessing and differentially expression analysis

Raw reads were screened to remove low quality reads, according to the following filter criteria: 1) reads with adaptor sequences. 2) the percentage of unknown bases (N) is greater than 10%. 3) percentage of the low quality bases (quality value ≤ 5) is greater than 50%. Statistics analysis and evaluation of data were performed, including total raw reads, total clean reads, Q20, Q30 and GC content. RSeQC software was used to quality-evaluate of all the data of RNA-Seq [27]. All downstream analyses were based on the quality-control clean reads. To evaluate the variation in gene expression, clean reads were mapped to the maize reference genome (B73 RefGenv3) using Bowtie version 2.2.4 [28] and TopHat version 2.0.13 [29], which utilize a quality and splice site aware alignment algorithm. The minimum and maximum intron length was set to 5bp and 60,000bp respectively, and all other parameters were set to the default values. Fragments per kilobase pair of exon model per million fragments mapped (FPKM) was used to normalize gene expression values and determined using Cufflinks version 2.2.1 [30]. SCC was calculated by Cor.test functions in R and used to quantify the reproducibility of data between the biological replicates of *rtcs* mutant and wild-type.

After calculating gene expression levels, the Cuffdiff [30] program within Cufflinks was used to detect genes differentially expressed between different samples. Cuffdiff measures variability of read counts for each gene across the replicates and uses these variance estimates to calculate the significance of observed changes in expression. Cuffdiff uses a corrected p-value, known as the q-value to determine if the differences between the two groups are significant (q-value<0.05). We used CummeRbund package in R to manage, visualize and integrate the results of the RNA-Seq analysis.

### Identification of co-expression clusters and cis-regulatory motifs

The SOTA function in the clValid package was used to identify co-expression clusters of highly correlated genes based on their expression profiles across four time points. Differentially expressed genes at least one comparison group were selected to construct co-expression clusters. The promoter region was defined as 2 kb upstream from the ATG start codon of co-expressed genes, and extracted by RSAT [31]. Motif discovery program oligo-ananlysis was used to identify candidate cis-regulatory motifs in the promoter regions of genes in each co-expression cluster [31]. Using the TOMTOM [32] motif comparison tool, the resulting motifs were compared against known motifs in the JASPAR CORE (2014) Plants database [33] to detect significantly similar known cis- regulatory motifs (q-value < 0.05).

### Gene annotation, functional enrichment and pathway enrichment analysis

For each differentially expressed gene, Gene Ontology (GO) annotation was obtained using the web-based agriGO tool [34]. Singular enrichment analysis (SEA) was used to GO enrichment analysis by comparing the list of differentially expressed genes to all expressed genes. A typical cut-off value of FDR < 0.05 was used in multiple comparison correction process. KOBAS2.0 software was used to test the statistical enrichment of DEGs in KEGG pathways [35]. Mapman software is a user-driven tool that displays large genomics datasets onto diagrams of metabolic pathways or other processes [36]. We utilized Mapman to assign DEGs into metabolic pathways in this study.

### Quantitative real-time PCR validation

The expression levels of 8 DEGs were determined by quantitative real-time reverse transcription PCR (qRT-PCR) with the corresponding primers (S2 Table). qRT-PCR was performed using the SYBR premix Ex Taq kit (TaKaRa, China) according to the standard protocol of the ABI 7500 Real-Time System (Applied Biosystems), as follows: 95°C for 2min; 95°C for 15s, 60°C for 1min, 40 cycles, and then generated the melt curves for verification of amplification specificity by a thermal denaturing step. We set all reactions in triplicate, as well as non-template controls. The 18S rRNA was used as an internal control for qRT-PCR amplification. The relative quantitative method (2^-△△CT^) was used to qunatify the expression levels of tested genes [37].

## Results

### Morphological and physiological characterization of root development in the wild-type and the mutant *rtcs*

**Fig 1:**
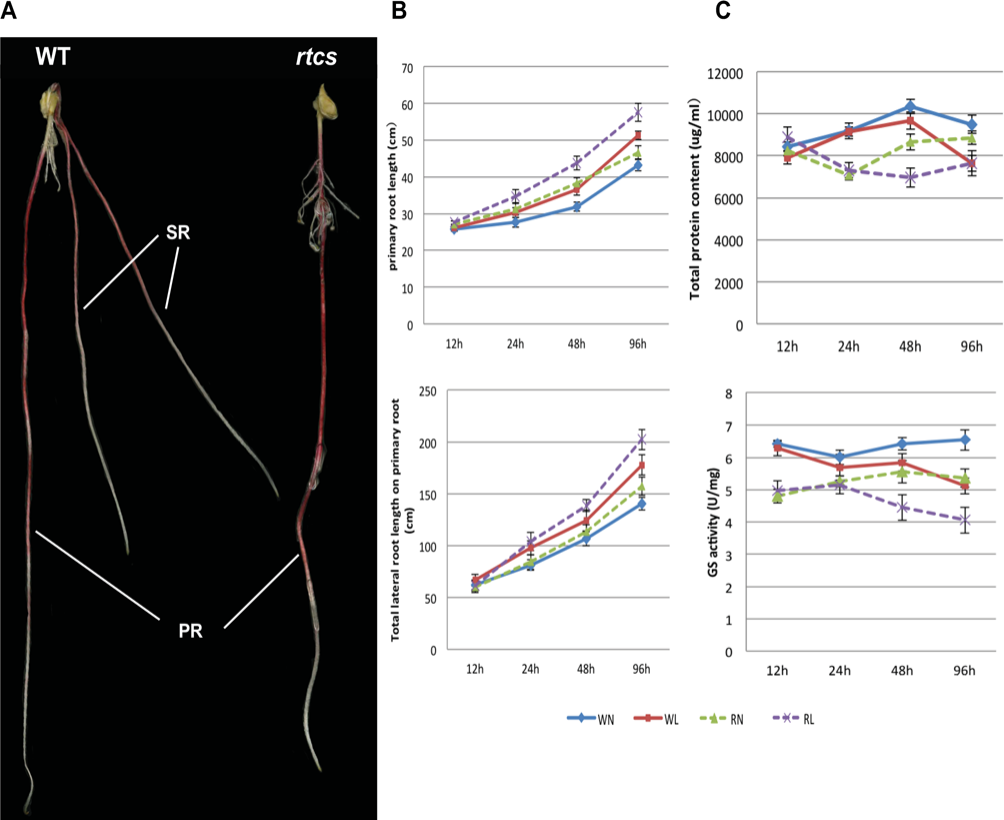
Morphological and physiological characterization of maize wild-type and *rtcs* after two N treatments. (A) The wild-type and *rtcs* root system at 8 d after germination. PR, primary root; SR, seminal root. (B) Morphological changes in PRL and LRL in maize wild-type and *rtcs*. (C) The total protein content and GS activity of the root system in both genotypes for different time points.

The root morphology of wild-type and *rtcs* seedlings can be distinguished (Fig 1) at 8 d after germination. The seminal root had been formed in wild-type seedlings but that was absent in *rtcs* seedlings (Fig 1A). Thus, we focus on the changes of primary and lateral root. The primary root length, total lateral root length on the primary root were compared between wild-type and *rtcs* seedlings at 12 h, 24 h, 48 h and 96 h after two N treatments. The differences between the two genotypes for both measurements were determined. Longer of PRL and LRL were observed in *rtcs* than wild-type on normal-N condition, and the difference in LRL was continuously increased with increasing time (Fig 1B). The increase in PRL and LRL was fairly smooth over time in both genotypes after low-N treatment, while larger changes of LRL were detected in *rtcs* (Fig 1B). The quantification of total protein content and GS activity revealed physiological differences between the *rtcs* mutant and wild-type under normal-N condition. The significant inhibitory effect of low-N stress on total protein content and GS activity appeared at 48 h after low-N treatment (Fig 1C). The total protein content and GS activity fluctuated with the stress time, but reductions were induced by low-N stress in both genotypes (Fig 1C).

### Transcriptome sequencing of maize mutant *rtcs* and its wild-type

In total, 32 libraries were constructed from roots of the maize mutant *rtcs* and its wild-type under low-N and normal-N conditions. Transcriptome sequencing of the libraries produced 34.1 to 47.5 million paired-end 125-bp reads corresponding to more than 5.0 billion nucleotides per sample (S3 Table). We obtained 32–46 M clean reads from raw data after quality checking and filtering (S3 Table). High quality scores were obtained for the reads, with Q20 percentages (percentage of sequences with sequencing error rate of <1%) greater than 92% (S3 Table). The GC contents of mutant *rtcs* and wild-type were similar (S3 Table). Between 78.82% and 85.57% of the remaining reads could be mapped onto the maize B73 reference genome sequence (S4 Table), with most of the reads mapping to exons (over 95%) and only a small proportion mapping to introns and intergenic regions.

Gene expression levels were quantified and reported as fragments per kilobase of transcript per million mapped reads (FPKM), estimated using Cufflinks [30]. Across the 32 samples, 27,921–28,547 known protein-coding genes were detected (S5 Table). In total, 25,285 expressed genes were common among all experimental time points and conditions. A pairwise Spearman’s correlation coefficient (SCC) analysis revealed high correlations between biological replicates (*r* = 0.973 to 0.986, S1 Fig), indicating that the sequencing data were reliable.

#### Dynamic shift in transcriptome levels during root development reveals an rtcs-dependent regulatory network

**Fig 2:**
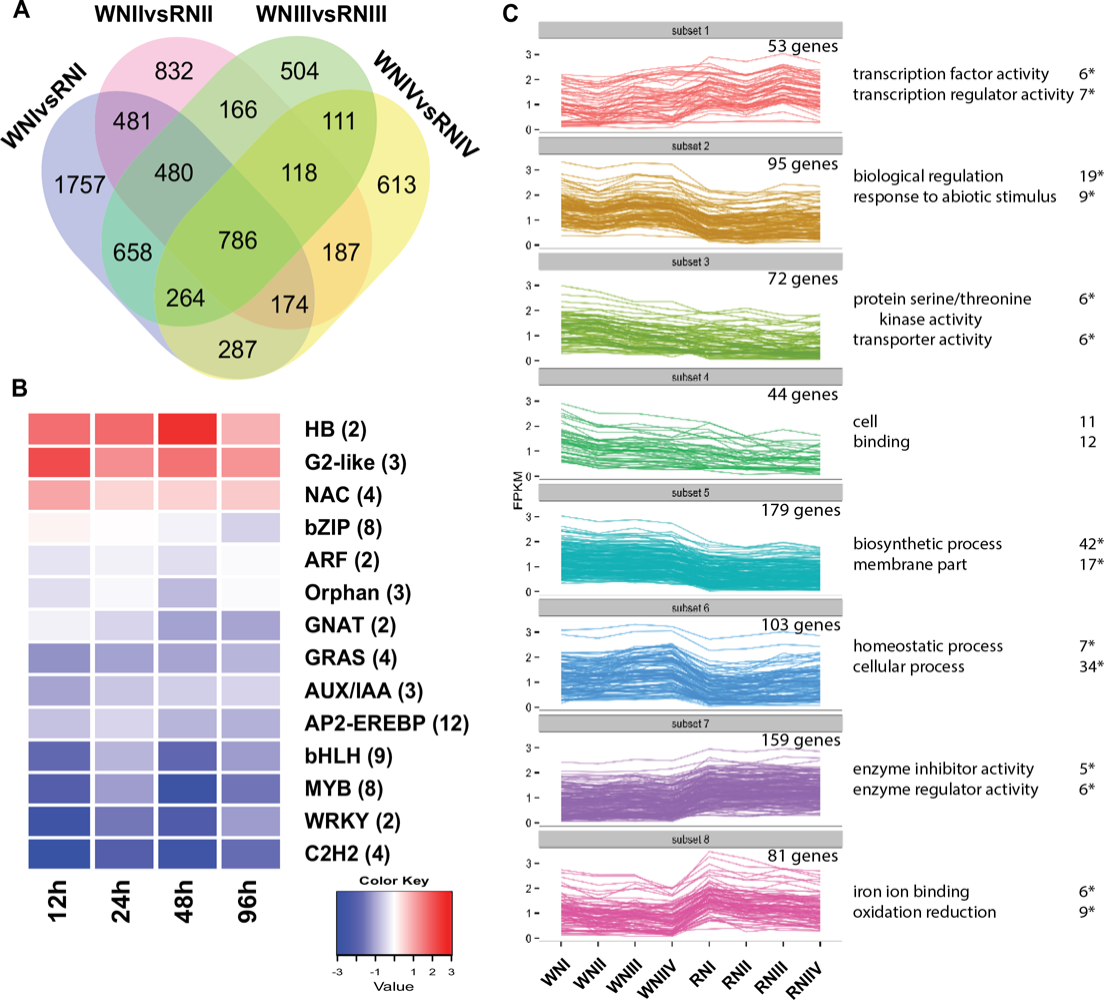
RNA-Seq analysis of *rtcs* and wild-type roots transcriptome under normal-N condition. (A) Venn diagram analysis of DEGs between wild-type and *rtcs* at four developmental time points. (B) Co-modulated differential expression transcription factors (TFs) between wild-type and *rtcs* at four time points. TFs were grouped by family and the number of differential expression TFs is indicated. Each column represents average expression differences across the TF family at one time point. (C) Clustering co-modulated DEGs based on the expression profiles (FPKM values were log10-transformed). The top GO terms and corresponding DEGs number are shown on the right side, “*” represents significant enrichment (FDR <0.05).

To gain insight into the regulatory network of root development, we analyzed differences in gene expression between the roots of mutant *rtcs* and roots of wild-type under normal-N conditions. In total, 7418 DEGs were revealed using Cuffdiff (Fig 2A, S6 Table). Among these genes, only 10.5% were detected in all comparison groups across four time points. The large number of time-specific DEGs indicated a dynamic shift during root development that might be regulated by *rtcs*. Next, we focused on co-modulated DEGs, which were more likely to play crucial roles in rtcs-dependent regulatory networks in maize. Based on KEGG annotations, the co-modulated DEGs were categorized into 79 pathways (S2 Fig). The main pathways were plant hormone signal transduction, phenylpropanoid biosynthesis, phenylalanine metabolism, glutathione metabolism, plant-pathogen interaction, starch and sucrose metabolism, galactose metabolism, and glycerolipid metabolism. Co-modulated DEGs were further functionally classified according to Gene Ontology (GO) terms using agriGO, and 29 significantly enriched GO terms were identified, such as biological regulation, regulation of primary metabolic process, regulation of transcription, regulation of gene expression, and response to abiotic stimulus (S7 Table). Between *rtcs* and wild-type, the transcript levels of genes encoding transcription factors (84 transcription factors in 32 transcription factor families) showed significant differences during the four time points of root development (S8 Table). In general, 12 AP2-EREBP, nine bHLH, and eight MYB transcription factor genes showed down-regulated expression in *rtcs* compared with wild-type during root development (Fig 2B, S8 Table). In contrast, genes encoding HB, G2-like, and NAC transcriptional factor families were up-regulated in *rtcs* compared with wild-type during root development.

To explore the significance of differences in gene expression profiles between the mutant *rtcs* and wild-type, the co-modulated DEGs were analyzed using the k-means clustering algorithm. As shown in Fig 2C, 786 co-modulated DEGs were divided into eight distinct clusters associated with various GO categories. For example, subset 1 included 53 genes that were overrepresented in transcription factor activity and transcription regulator activity, and subset 2 contained 95 genes that were significantly enriched in biological regulation and response to abiotic stimulus. Remarkably, there were 62 genotype-specific genes with unclear functions that were only expressed in wild-type (S6 Table). We extracted upstream gene sequences from these genotype-specific genes to detect putative cis-regulatory elements. Of these 62 genes, 23 contained the LBD motif, which might be directly regulated by *rtcs* (S9 Table).

**Fig 3:**
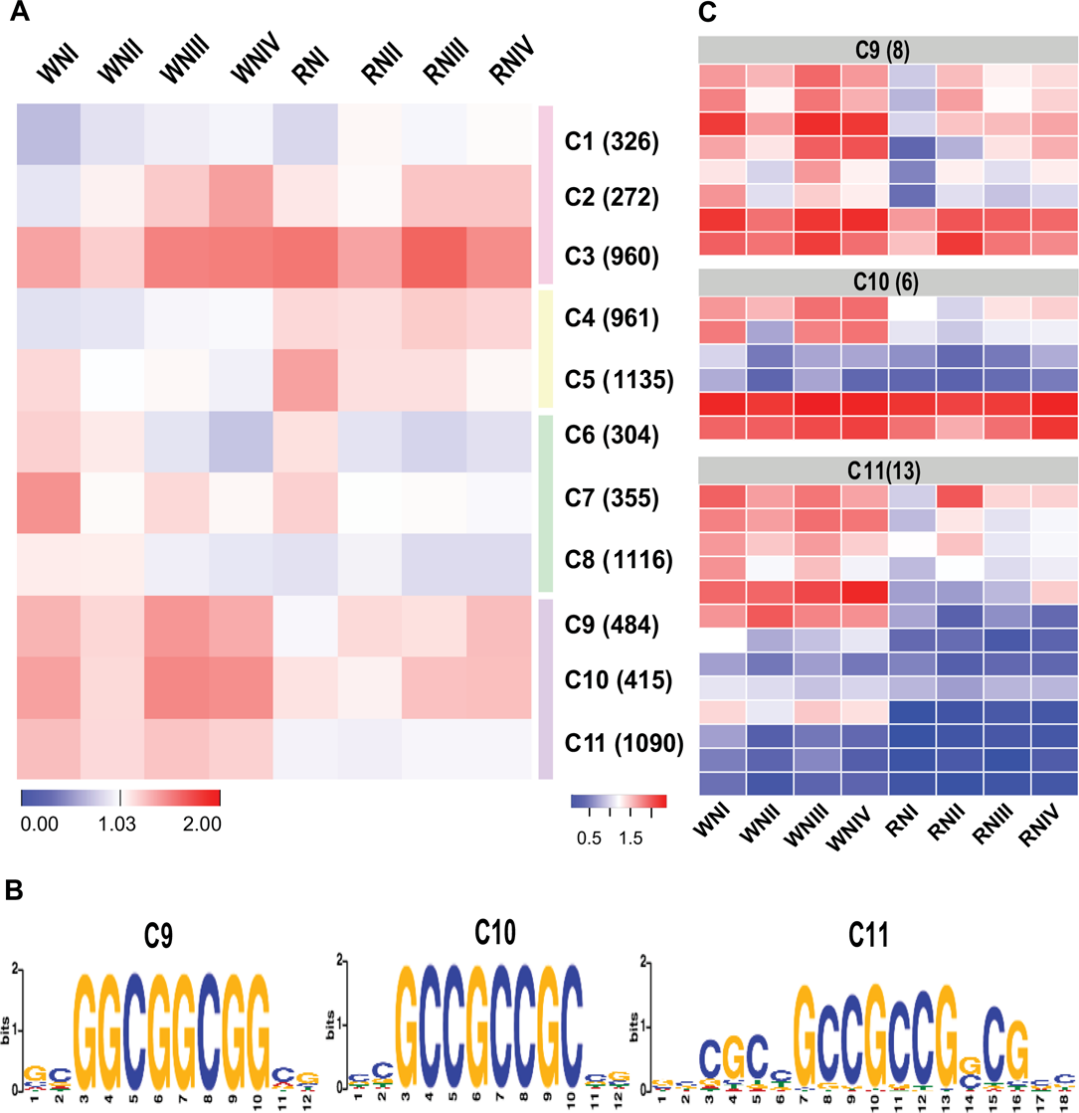
Construction of rtcs-dependent regulatory networks of root development. (A) Expression signatures across wild-type and *rtcs* libraries under normal-N condition were used to cluster genes with dynamic expression during root development. Eleven co-expression clusters fell into four distinct clades of expression, cluster 9, 10 and 11 were highly enriched in wild-type. Each cluster is assigned a number identifier and the number of genes associated with each cluster is indicated (right). The heatmap represents cluster centers (FPKM values were log10-transformed). (B) De novo cis-regulatory motifs overrepresented in the promoter regions of the genes in co-expression cluster 9, 10 and 11. Three cis-regulatory motifs were obtained with significant similarity to the reported binding sites of the AP2-EREBP superfamily. (C) Expression analysis of 27 AP2-EREBP genes in wild-type and *rtcs*. The number of AP2- EREBP family members is shown at the top.

To characterize gene expression signatures associated with the rtcs-dependent regulatory network involved in root development, all significant DEGs were grouped based on their dynamic expression profiles across four time points. Using the SOTA clustering approach, the 7418 DEGs were divided into 11 distinct co-expression clusters representing the features of developmental change (Fig 3A). Co-expression clusters 9, 10, and 11 were highly enriched in wild-type (Fig 3A), suggesting that these genes were closely associated with the rtcs-dependent regulatory network. These clusters were further analyzed to explore their rtcs-dependent co-expression regulatory networks by searching for cis-regulatory motifs. We searched for candidate motifs overrepresented within the promoter regions of genes in each cluster, and identified motifs with significant similarity to other known plant cis-regulatory motifs. In the three co-expression clusters, we detected three cis-regulatory motifs showing significant similarity to the reported binding sites of ABI4 (MA0123.1) and ERF1 (MA0567.1, Fig 3B). Both ABI4 and ERF1 belong to the AP2-EREBP superfamily, whose members regulate diverse processes in plant development and stress responses, such as vegetative and reproductive development, cell proliferation, hormonal signal transduction, and responses to biotic and abiotic stresses [38]. The consensus finding was that the three co-expression clusters included 27 AP2-EREBP genes (Fig 3C). This result suggested that members of the AP2-EREBP family play broad regulatory roles in the rtcs-dependent regulatory network in maize root development. As shown in the heat map, the transcript levels of genes encoding AP2-EREBP transcription factors differed between wild-type and *rtcs;* that is, their average FPKM values were higher in wild-type than in *rtcs* (Fig 3C).

To identify the major biological functions associated with the co-expression clusters, SEA and SEACOMPARE were used to identify the processes that were significantly enriched. The specific co-expression clusters were associated with specific biological processes and molecular functions including multicellular organismal process, regulation of biological process, cell death, and N compound metabolic process (S3 Fig). These results suggested that co-expression clusters had distinct functions. Further detailed analyses revealed that these co-expression clusters also contained other genes without functional annotations, which warrant further investigation.

### Transcriptional changes in *rtcs* and wild-type under N deficiency

To gain a global view of the N-deficiency-responsive regulatory networks in maize roots, we made eight pairwise comparisons between the mutant *rtcs* and wild-type roots under low-N and normal-N conditions. Comparison of transcript abundance revealed 7977 and 7762 N-deficiency-responsive DEGs in wild-type and *rtcs*, respectively (S4A and S4B Fig). Of those identified in the wild-type, 194 genes were up-regulated and 399 genes were down-regulated across four time points under N-deficiency stress. Among them, 31 genes were very strongly induced by N-deficiency in wild-type (>eight-fold induction, S10 Table), including geranylgeranyl pyrophosphate synthase (GRMZM2G058404), splicing factor U2af subunit isoform (GRMZM2G031827), isocitrate lyase (GRMZM2G056369), beta-glucanase (GRMZM2G473711), glycosyltransferase (GRMZM2G105221), and aldose 1-epimerase (GRMZM2G125233). In contrast, *rtcs* showed larger dynamic changes in gene expression under N-deficiency stress, and 804 co-modulated DEGs were detected (S10 Table). More N-deficiency-induced genes were detected in *rtcs* than in wild-type. In *rtcs*, 543 DEGs were induced and 261 DEGs were repressed under N-deficiency stress (S10 Table). Some functional genes encoding stress and defense responses related proteins, transporters and transcription factors were up-regulated with above ~eight-fold change in *rtcs* under N-deficiency stress, such as cytochrome P450 superfamily proteins (GRMZM2G399530, GRMZM2G129860), glucose-6-phosphate/phosphate translocator (GRMZM2G009223), SWIb domain-containing protein (GRMZM2G077088), ZIM motif family protein (GRMZM2G173596), V-type proton ATPase 16 kDa proteolipid subunit (GRMZM2G028432), receptor protein kinase TMK1 (GRMZM2G001934), R2R3MYB-domain protein (GRMZM2G104789), MYB DNA-binding domain superfamily proteins (GRMZM2G425427, GRMZM2G001824) and AP2-EREBP transcription factor superfamily proteins (GRMZM2G020054, GRMZM2G052667).

**Fig 4:**
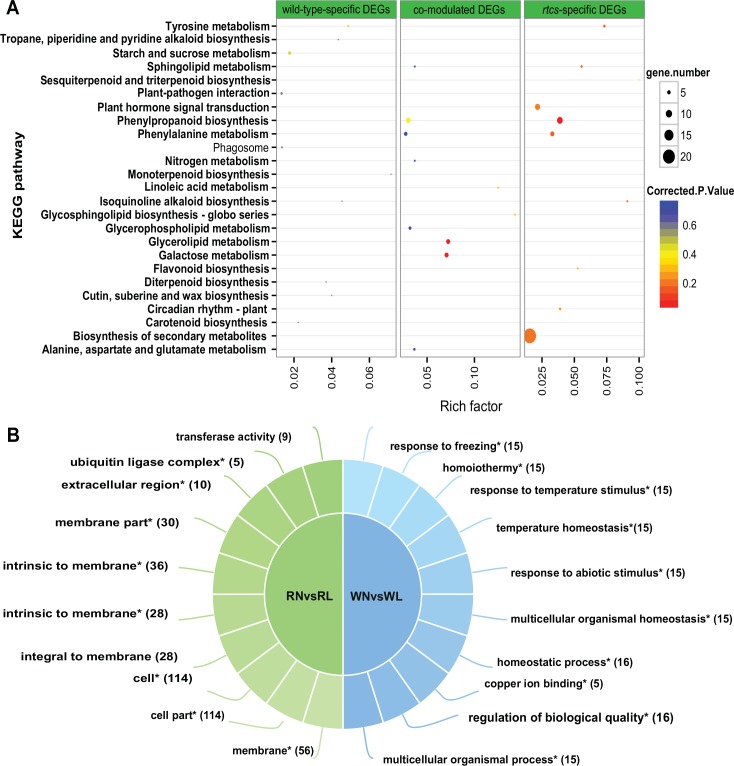
Gene annotation and functional enrichment analysis for DEGs in response to N-deficiency. (A) Cross-comparison of pathway enrichment analysis among DEGs in response to N-deficiency. The *y*-axis corresponds to the KEGG pathway, and the *x*-axis shows the enrichment factor. The color of the dot represents the *q*-value, and the size of the dot represents the number of DEGs mapped to the reference pathways. (B) Cross-comparison of enriched GO terms among DEGs in response to N-deficiency. The top GO terms and corresponding DEGs number are shown on the right side, “*”represents significant enrichment (FDR <0.05).

Although many genotype-specific transcriptional changes were found, 403 genes were co-modulated in both genotypes across four time points under N-deficiency stress (S4C Fig, S10 Table). To gain insight into the effects of N-deficiency on gene transcription profiles, co-modulated DEGs in both genotypes were assigned to GO categories consisting of 90 biological processes, 72 molecular functions, and 23 cellular components (S5 Fig). In the biological processes category, metabolic process (151 DEGs) and cellular processes (115 DEGs) were strongly represented, indicating that important cellular and metabolic processes occurred in maize roots under N-deficiency stress. In the molecular functions category, 151, 112, and 48 co-modulated DEGs were identified as belonging to binding, catalytic activity, and ion binding subcategories, respectively. To analyze the biological functions of co-modulated DEGs, pathway enrichment analysis using KOBAS was implemented. This analysis identified two significantly enriched pathways under N-deficiency; glycerolipid metabolism and galactose metabolism (S11 Table, Fig 4A). In addition, seven and five co-modulated DEGs were annotated with the phenylpropanoid biosynthesis and phenylalanine metabolism pathways, respectively (S11 Table).

Analyses of the specific transcriptional changes induced under N-deficiency stress revealed 190 and 401 genes that were differentially regulated in wild-type and *rtcs*, respectively, across four time points (S4C Fig). The functional differences between the two genotypes were explored using functional annotation and enrichment analyses. Compared with *rtcs*, wild-type had a larger portion of genotype-specific DEGs that were significantly enriched in the following GO terms: response to freezing, response to abiotic stimulus, multicellular organism homeostasis, copper ion binding, regulation of biological quality, and multicellular organism process (Fig 4B). Correspondingly, many of the *rtcs*-specific DEGs were classified in the following cellular component subcategories: membrane, cell, membrane part, extracellular region, and ubiquitin ligase complex (Fig 4B). Nine *rtcs*-specific DEGs were classified in the phenylpropanoid biosynthesis pathway subcategory (S11 Table, Fig 4A). A few genes were expressed in only one genotype; further research on the function of these genes is warranted. Together, these results suggested that the differential expression of genes in wild-type and *rtcs* may contribute to the differences in the regulatory mechanisms of N-deficiency between the two genotypes.

Real-time RT-PCR analyses were conducted to evaluate the transcript levels of eight representative responsive DEGs involved in different biological processes (response to stress, oxidation-reduction process, ion binding, and anatomical structure development). The results of these analyses confirmed that these genes were induced under N-deficiency stress. The gene transcript profiles were consistent with those predicted from the RNA-Seq data (*R*^*2*^ = 0.73, S6 Fig).

### DEGs associated with root development participate in response to N deficiency

**Fig 5:**
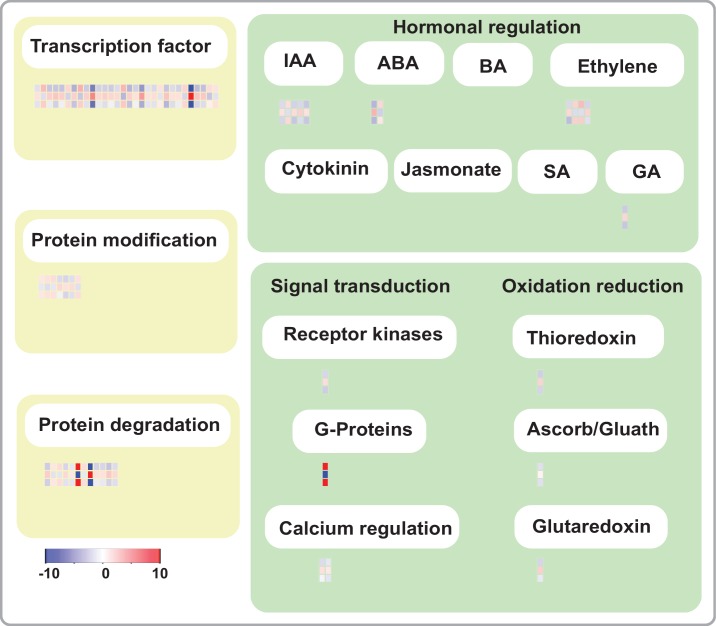
Gene annotation of co-modulated DEGs, analyzed and visualized by MapMan. Each block represents average expression changes for one gene. Three rows of blocks are shown to demonstrate wild-type-specific N-deficiency DEGs, *rtcs*-specific N-deficiency DEGs and rtcs-dependent common DGEs from bottom to top.

Plant roots are critical for N acquisition; therefore, genes controlling root development may also affect a plant’s responses to N stress. In a comparative analysis, we found that 609 DEGs associated with root development also responded to N deficiency. This high proportion of common DEGs suggested that many DEGs associated with root development also participate in the response to N deficiency. To understand and explore the role of the rtcs-dependent regulatory network in the N-deficiency response, we selected 245 co-modulated DEGs for functional annotation using MapMan software (S12 Table). These 245 genes were those that were common to the rtcs-dependent DEGs set and the genotype-specific N-deficiency DEGs set. The functional annotations included many types of biological regulation, including transcription regulation, protein modification, protein degradation, signal transduction, oxidation-reduction, and hormonal regulation (Fig 5, Table 1), and 189 (77.14%) of the 245 genes were assigned to more than one functional class (S12 Table). Previous studies have shown that transcription factors can affect the plant response to abiotic stress. AP2-EREBP, ARR, bHLH, C2C2-zinc finger, MYB, bZIP, and HB transcription factor families have been shown to participate in the response to N stress (Fig 5, Table 1). At the levels of protein modification and degradation, serine/threonine-protein phosphatases, calmodulin-dependent protein kinases, shaggy kinase homologs, leucine-rich repeat transmembrane protein kinases, endopeptidases, and ubiquitin-protein ligases were differentially expressed at various levels of significance. The ARF protein, HVA22-like protein, and members of the AP2-EREBP transcription factor family also participated in the response to N-deficiency and in root development. In addition, DEGs related to signal transduction and oxidation-reduction showed dynamic expression in maize roots in response to N-deficiency stress.

**Table 1:** Co-modulated DEGs associtated with root development and response to N stress were involved in many levels of biological regulation.

gene_id	gene_ description	regulatory_methods	expression_ ratios*		
			WLvsWN	RLvsRN	RNvsWN
GRMZM2G102059	AP2-EREBP	Transcription regulation	-1.08	1.13	-1.13
GRMZM2G544539	AP2-EREBP	Transcription regulation	1.95	-1.24	2.61
GRMZM2G160971	AP2-EREBP	Transcription regulation	-0.73	1.97	-2.20
GRMZM2G069146	AP2-EREBP	Transcription regulation	-1.78	2.23	-2.21
GRMZM2G138396	AP2-EREBP	Transcription regulation	-0.63	1.95	-2.28
GRMZM2G479110	ARR	Transcription regulation	1.46	-1.71	1.63
GRMZM2G082343	bHLHBasic	Transcription regulation	-2.29	1.82	-3.00
AC193786.3_FG005	bHLHBasic	Transcription regulation	1.93	-2.11	2.98
GRMZM5G849600	bHLHBasic	Transcription regulation	-1.11	1.80	-2.00
AC197764.4_FG003	C2C2(Zn)	Transcription regulation	-5.51	4.67	-4.85
GRMZM2G069176	C2H2	Transcription regulation	-0.78	1.57	-1.81
AC185108.3_FG011	C2H2	Transcription regulation	-1.24	1.88	-1.90
GRMZM2G113860	C2H2	Transcription regulation	-0.54	1.59	-1.98
GRMZM2G139024	E2F/DP	Transcription regulation	-1.43	1.50	-1.39
GRMZM2G117164	HBHomeobox	Transcription regulation	2.09	-2.72	2.72
GRMZM2G169356	MYB	Transcription regulation	-2.38	1.78	-2.79
GRMZM2G047600	MYB	Transcription regulation	-0.55	1.18	-1.82
GRMZM2G425427	MYB	Transcription regulation	-3.27	3.89	-3.95
GRMZM2G088140	bZIP	Transcription regulation	-0.98	1.19	-1.05
GRMZM2G444748	bZIP	Transcription regulation	-1.09	1.33	-1.50
GRMZM2G445575	bZIP	Transcription regulation	0.75	-1.10	1.13
GRMZM2G079185	LBD	Transcription regulation	-2.78	2.25	-2.21
GRMZM2G004988	SSXT protein	Transcription regulation	-1.34	1.24	-1.43
GRMZM2G303964	ubiquitin-protein ligase	Transcription regulation	-0.66	1.20	-1.72
GRMZM2G104299	AT-hook protein	Transcription regulation	1.63	-1.60	1.69
GRMZM2G126106	Remorin	Transcription regulation	WN	RL	WN
GRMZM2G175927	aspartyl protease family protein	Transcription regulation	-1.58	2.23	-2.54
GRMZM2G468657	Aspartic proteinase nepenthesin-1	Transcription regulation	-1.33	2.05	-2.41
GRMZM2G137352	Remorin	Transcription regulation	0.58	-2.21	1.36
GRMZM2G461793	MAK16 protein-related	Transcription regulation	1.18	-1.05	1.05
GRMZM2G062394	Serine/threonine-protein phosphatase	Protein modification	0.95	-0.90	0.95
GRMZM2G422576	calmodulin-dependent protein kinase	Protein modification	1.17	-1.26	1.11
GRMZM2G035996	Shaggy kinase homolog	Protein modification	1.18	-0.94	1.33
GRMZM2G041774	protein kinase	Protein modification	-0.56	1.51	-1.44
GRMZM2G063897	protein kinase	Protein modification	-1.65	1.14	-1.62
GRMZM2G158045	protein kinase	Protein modification	-1.20	1.40	-1.30
GRMZM2G059497	leucine-rich repeat transmembrane protein kinase	Protein modification	1.27	-1.16	1.23
GRMZM5G839014	OTU-like cysteine protease family protein	Protein degradation	-1.87	1.97	-1.91
GRMZM2G013461	Multidomain cystatin	Protein degradation	0.95	-1.10	1.02
GRMZM2G006377	cysteine-type endopeptidase	Protein degradation	1.37	-1.01	1.38
GRMZM2G055698	serine-type endopeptidase	Protein degradation	-1.06	1.47	-1.59
GRMZM2G066996	serine protease inhibitor	Protein degradation	-0.99	0.91	-0.92
GRMZM2G042782	ubiquitin interaction motif-containing protein	Protein degradation	WL	RN	RN
GRMZM2G010460	ubiquitin-protein ligase	Protein degradation	1.30	-0.87	1.28
GRMZM2G440918	ubiquitin-protein ligase	Protein degradation	WN	RL	WN
GRMZM2G303964	ubiquitin-protein ligase	Protein degradation	-0.66	1.20	-1.72
GRMZM2G055052	U-box domain-containing protein	Protein degradation	-0.78	1.15	-1.64
GRMZM2G082653	Zinc finger-like protein	Protein degradation	-1.72	2.08	-2.28
GRMZM2G156490	F-box domain containing protein	Protein degradation	-1.27	1.50	-1.32
GRMZM2G080565	O-fucosyltransferase family protein	hormona regulation	-1.63	1.13	-1.43
GRMZM2G134156	growth regulator like protein	hormona regulation	1.47	-1.17	1.30
GRMZM2G361993	auxin-responsive family protein	hormona regulation	-2.14	1.42	-1.77
GRMZM2G365162	auxin-responsive family protein	hormona regulation	-1.31	1.78	-1.54
GRMZM2G066219	auxin-responsive family protein	hormona regulation	-2.11	1.20	-2.12
GRMZM2G092925	HVA22-like protein	hormona regulation	-2.77	2.89	-2.81
GRMZM2G154735	HVA22-like protein	hormona regulation	0.93	-1.82	1.57
GRMZM2G110369	oxidoreductase	hormona regulation	-2.57	1.56	-1.49
GRMZM2G020150	AP2-EREBP	hormona regulation	1.82	-1.35	1.67
GRMZM2G544539	AP2-EREBP	hormona regulation	1.95	-1.24	2.61
GRMZM2G123119	AP2-EREBP	hormona regulation	-1.15	1.66	-1.65
GRMZM2G044481	Ent-copalyl diphosphate synthase	hormona regulation	-2.16	1.63	-1.99
GRMZM2G318942	RAB GDP-dissociation inhibitor	signal transduction	WL	RN	RN
GRMZM2G061723	calmodulin-binding family protein	signal transduction	-0.42	1.19	-1.36
GRMZM2G096228	Caltractin	signal transduction	-1.02	0.87	-0.93
GRMZM2G307262	protein disulfide isomerase	oxidation reduction	-1.71	1.69	-1.90
GRMZM5G828229	monodehydroascorbate reductase	oxidation reduction	-1.14	0.45	-1.19
GRMZM2G023237	Grx_I1-glutaredoxin subgroup III	oxidation reduction	-0.83	1.78	-1.59

* Expression ratios were represented by the average of expression differences across four time points. All ratios are presented as log2 fold change. Sample name indicate that the gene only expressed in corresponding sample.

## Discussion

### Root morphological and physiological variation contributed to the responses to low-N stress

It has been proposed that morphological and physiological plasticity of plant roots play vital roles in response to N stress. The general response of maize roots to N deficiency is to optimize root system architecture, including root length, root number, and growth angles [15]. The root system of maize comprises embryonic root and post-embryonic root systems. Among them, primary, seminal, crown and brace roots constitute axile roots, whereas lateral roots arise from axile roots [39]. An increased investment in the diameter and length of primary roots would be beneficial for plant anchorage and access to deep or highly mobile resources [15]. The primary lateral roots also play an important role in plant anchorage and mobile and immobile resources acquisition by providing additional root surface area [15]. Here, we use mutant *rtcs* and wild-type to investigate the mechanism in response to low-N stress in maize root. We focus on the alterations of PRL and LRL on the primary root. The differences between the two genotypes were determined at 12 h, 24 h, 48 h and 96 h after two N treatments. The common feature that PRL and LRL increased after low-N treatment was observed in both genotypes (Fig 1B), which indicated that low-N stress induced positive responses in maize root. These results are consistent with the findings of previous study [40]. However, the positive effect of low-N stress on lateral growth might alter to negative effect, when the stress prolonged and seriously affected root growth [40]. At physiological levels, GS plays an important role in increasing N acquisition. The inhibitory effect of low-N stress on total protein content and GS activity was observed in both genotypes. These results agree with the previous study. Noteworthy, larger changes of LRL were detected in *rtcs*. The mutant *rtcs* tended to increased N uptake under low-N stress by compensatory growth of lateral roots. The axial roots elongation and lateral root growth are main factors that contributed to N uptake in maize under N deficiency [41, 42]. It has been reported that increased N uptake in maize plants after root growth restriction is mainly achieved by morphological variation of the remaining roots rather than by increased root N uptake rate [43]. Considering the above, the primary roots elongation and lateral root compensatory growth are main response strategies to offset the deficiency of seminal and shoot-borne roots in *rtcs* under N-deficiency.

### The rtcs-dependent regulatory networks play vital roles in root development

Maize has a complex root architecture with several different types of roots. Recently, several genes controlling maize root development have been isolated. Among them, *rtcs* was shown to play a key role in the auxin-mediated initiation of seminal and shoot-borne roots in maize [14]. In this study, we explored differences in gene expression patterns between *rtcs* and wild-type roots at four time points during root development. We identified 786 co-modulated DEGs that were involved in diverse processes, including plant hormone signal transduction, glutathione metabolism, galactose metabolism, starch and sucrose metabolism, phenylpropanoid biosynthesis, and phenylalanine metabolism. Twenty-three out of 62 wild-type-specific genes with unclear function contained the LBD motif, suggesting that they might be regulated by *rtcs* (S9 Table). NF-YA transcription factors, belonging to the CCAAT-box binding factor family, play roles in a wide range of plant growth and development processes [44]. In the present study, we found that NF-YA transcription factor (GRMZM2G026157) contained the LBD motif and completely abolished in mutant *rtcs* (S9 Table). These wild-type-spedific genes should be studied in more detail using genetic analyses in future research.

LBD proteins control root development by a specific and conserved mechanism––Aux-LBD module [45]. In Arabidopsis, LBD16/18/29 combinatorially regulated lateral root formation as direct regulatory targets of AUX/IAA-ARF modules [46]. The *rtcs* gene is the closest homolog of *AtLBD16/29* in maize [19]. Loss of function mutation in this gene resulted in reduced expressions of ARF [47] and AUX/IAA (3) transcription factors (Fig 2B). In addition, nine bHLH transcription factor genes showed down-regulated expression in *rtcs* compared with wild-type during root development. It has been previously reported that the members of the bHLH transcription factor family were capable of interacting with LBD proteins by post-translational regulatory [22].

The AP2-EREBP family is a large group of plant-specific transcription factors that contain the highly conserved AP2 DNA-binding domain [48]. These proteins are involved in a variety of pathways that regulate plant growth and development, and play multiple roles in responses to different biotic and abiotic stresses [49, 50]. The AP2-EREBP family has been shown to control root, flower, stalk, and leaf development [51–54]. The maize AP2-EREBP gene *ZmRap2*.*7* has been proved to be a negative regulator of flowering time [55]. The branched silkless1 (*bd1*) is preferentially expressed in wild-type coleoptile nodes during maize root development, implying that *bd1* plays a role in the formation of crown roots [53]. PLETHORA2 in Arabidopsis is another AP2-EREBP transcription factor required for the formation of root stem cells and roots [54]. The closest maize homolog of PLETHORA2 is baby boom1, which was almost exclusively expressed in the wild-type coleoptile node but not in that of the *rtcs* mutant [53]. In addition, AtERF71/HRE2 and AtERF73/HRE1 are known to affect root cell expansion and cell division, respectively, to increase PRL [51]. In the present study, 12 AP2-EREBP transcription factors tended to show down-regulated expression in *rtcs* compared with wild-type across four time points (Fig 2B, S8 Table). Co-expression clustering analysis revealed that co-expression clusters 9, 10, and 11 were strongly associated with the rtcs-dependent regulatory network (Fig 3A). The cis-regulatory motif analysis of co-expression clusters revealed that clusters 9, 10, and 11 were regulated by AP2-EREBP transcription factors, and 27 AP2-EREBP genes were discovered among these three clusters. The previous research revealed that Arabidopsis AP2-EREBP transcription factor PUCHI co-acted with LBD16 and LBD18 downstream of the AUX/IAA-ARF7/19 module in lateral root development [52, 56]. These results indicated that the AP2-EREBP family might play broad regulatory roles in the rtcs-dependent regulatory network in maize root development. Taken together, these results suggest that rtcs-dependent regulatory network regulate root development through AUX–LBD module in auxin signaling pathway. Some transcription factors as co-act factors or interaction factors also participate in regulatory network of root development, such as NF-YA, bHLH, AP2-EREBP.

### Regulatory network of root development controls gene expression in response to N deficiency

To gain a deeper understanding of the complex regulatory mechanisms controlling the response to N deficiency, we used a comparative RNA-Seq-based approach to compare gene expression profiles of *rtcs* and wild-type roots under low-N and normal-N conditions. These analyses showed that the genes regulating the responses to N deficiency had broad functional classifications. Under low-N conditions, there were marked changes in the transcript levels of genes involved in glycerolipid metabolism, galactose metabolism, phenylpropanoid biosynthesis, and the phenylalanine metabolic pathway. These results provided a deeper insight into the N-deficiency response in maize. There was also clear evidence that N deficiency affected various secondary metabolic pathways, including the phenylpropanoid, flavonoid, and anthocyanin metabolic pathways [57]. DEGs related to phenylpropanoid metabolism were detected in the co-modulated DEGs set and in the genotype-specific DEGs set. Changes in the transcript levels of genes encoding transcription factors occurred during the early stage of the N-deficiency response [57]. Some functional genes encoding proteins and transcription factors related to stress and defense responses were significantly up-regulated in *rtcs*. These included genes encoding a cytochrome P450 superfamily protein, and members of the MYB and AP2-EREBP transcription factor families. The AP2-EREBP family is known to respond to N deficiency [58]. Here, seven and 10 genes encoding AP2-EREBP transcription factors showed dynamic changes in expression in the *rtcs* mutant and wild-type, respectively, under N-deficiency stress (S10 Table). Among them, two AP2-EREBP transcription factors were up-regulated at least eight-fold in *rtcs* under N deficiency.

During evolution, plants have evolved sophisticated physiological and morphological responses to balance the amount of N acquired for growth and development. These responses include the regulation of N absorption, adjustment of the shoot/root ratio, and changes in root architecture [6]. Thus, root development is closely related to N-stress responses. In this study, we found that a high proportion of DEGs associated with root development also participated in the response to N deficiency. To understand and explore rtcs-dependent regulatory networks under N-deficiency stress, we analyzed 245 co-modulated DEGs related to root development and N-deficiency responses in detail (S12 Table). Opposite changes in the transcript patterns of these DEGs were observed in *rtcs* and wild-type under low-N conditions, suggesting these co-modulated DEGs were mediated by rtcs-dependent regulatory networks. A functional annotation analysis revealed that these genes were involved in biological regulation at several levels; transcription regulation, protein modification, protein degradation, signal transduction, oxidation-reduction, and hormonal regulation (Fig 5, Table 1).

Transcriptional regulation and post-transcriptional modifications contribute substantially to plant growth and development and stress responses [38, 59]. There are 66 co-modulated DEGs were involved in many levels of biological regulation (Table 1). Among them, eight co-modulated DEGs were specific expressed in maize root, including GRMZM2G047600, GRMZM2G069146, GRMZM2G082343, GRMZM2G123119, GRMZM2G138396, GRMZM2G169356, GRMZM2G425427, GRMZM5G849600 [60] (Table 1). At the level of transcriptional regulation, 22 transcription factors were found to participate in the regulation of root development and in the response to N deficiency. The AP2-EREBP family was well represented, with five AP2-EREBP members showing differential expression patterns. In future studies, it will be interesting to analyze the roles of AP2-EREBP transcription factors in the N-deficiency responses and in root development. The activities of AP2-EREBP TFs were reported to be subject to post-transcriptional modification [38, 50]. We detected 19 genes related to protein modification and protein degradation among the DEGs under low-N conditions, such as serine/threonine-protein phosphatase, shaggy kinase homolog, endopeptidase and ubiquitin-protein ligase. Previous studies have shown that N stress induces profound changes in the expression of genes related to oxidative stress, and reactive oxygen species (ROS) signaling pathway via AP2-EREBP transcription factor may play an important role in adaptation to N deficiency [61, 62]. In this study, three of the DEGs under N-deficiency encoded oxidation-reduction proteins: protein disulfide isomerase, monodehydroascorbate reductase, and Grx_I1-glutaredoxin subgroup III.

Several recent reports have provided evidence that phytohormones such as auxin, abscisic acid, and ethylene (ETH) are associated with root development and N-stress responses [6]. In this study, 12 genes encoding proteins involved in hormonal regulation were identified, including AP2-EREBP proteins, ARF proteins, HVA22-like proteins, and ent-copalyl diphosphate synthase. It has been reported that auxin-responsive family proteins participated in early auxin response and nutrient allocation [63]. Three auxin-responsive family proteins were also identified in hormona regulation level by MapMan annotation. *ZmMDAR4* (GRMZM5G828229) were down-regulated under ABA treatment, and respond to abiotic stresses [64]. In the present study, *ZmMDAR4* showed down-regulated expression in wild-type under low-N treatment, and higher expression level were identified in wild-type than *rtcs* mutant. Ethylene regulates root growth by mediating IAA biosynthesis and transport machinery, and the members of AP2-EREBP family play roles in IAA- and ETH-signaling [6, 65, 66]. Overexpression of the AP2-EREBP transcription factor *GhERF12* in Arabidopsis activated a constitutive ethylene response related to IAA biosynthesis and/or signaling; consequently, the *GhERF12-*overexpressing plants showed excess endogenous IAA levels, which inhibited plant growth and development [65]. Based on those results, we speculated that AP2-EREBP transcription factors, which are key factors in multiple hormone signaling pathways, may ultimately regulate root development and the N-deficiency response.

In Arabidopsis, *LBD* genes mediate the repressive effect of N-responsive genes, including key genes required for N uptake and assimilation. Consequently, changes in the expression levels of *LBD*s affect N content, nitrate reductase activity/activation, levels of proteins, amino acids, and starch, and N-related growth phenotypes [62, 67]. In the present study, NRT2.1, NAR2.1, NAR2.2, and NAR2.3 showed much higher expression values in *rtcs* than in wild-type under normal-N conditions, indicating that *LBD* genes function mainly as transcriptional repressors, as noted in previous studies. However, a diversity of expression changes was observed in these nitrate transporters under low-N conditions, revealing the complexity of the rtcs-dependent regulatory networks. Nitrate transporters also control root development. For example, NRT1.1 was shown to act upstream of ANR1 in the signaling pathway, triggering lateral root growth and root colonization of NO_3_^-^-rich patches [68]. In addition, NRT2.1 was proposed to repress lateral root initiation in response to nutritional cues by acting either as a NO_3_^-^ sensor or signal transducer [69]. The LBD transcription factors are strong candidates for transcription factors that negatively regulate genes encoding nitrate reductase and other N assimilation-related proteins in response to changes in the N metabolic balance [67]. Further functional characterization of these LBD transcription factors will clarify their roles in the N responses of maize.

### Conclusions

A complex set of molecular regulatory networks regulate root development and N metabolism. The distinguishing morphological and physiological characteristics of nitrogen responses on root architecture were observed between the wild-type and the mutant *rtcs* of maize. In this study, we performed comparative RNA-Seq-based analyses to compare gene expression profiles between *rtcs* and wild-type roots under different N conditions. We identified 786 co-modulated DEGs that were related to root development and participated in important metabolic pathways. Analyses of co-expression clusters and cis-regulatory elements revealed the importance of the AP2-EREBP transcription factor family in the rtcs-dependent regulatory network. Comparative RNA-Seq analysis revealed differences in gene transcription between roots of *rtcs* and wild-type, and 403 co-modulated DEGs with distinct functions were detected. A comparative analysis revealed that the regulatory networks controlling root development also controlled gene expression in response to N deficiency. These comprehensive analyses revealed that several AP2-EREBP family members play important roles in the rtcs-dependent regulatory network related to both root development and the N-deficiency response. Taken together, this information will increase our understanding of the molecular regulatory networks controlling root development and N-stress responses.

## Supporting Information

S1 Fig SCC analysis of the transcriptome data for biological replicates using log10-based FPKM values. (PDF)

S2 Fig KEGG pathway annotation of co-modulated DEGs between wild-type and *rtcs* under normal-N condition. The y-axis corresponds to KEGG Pathway, and the x-axis shows DEGs number. The color of the dot represent enrichment factor.
(PDF)

S3 Fig Cross-comparison of enriched GO terms among co-expression clusters by single enrichment analysis (SEA). Different colors in the block represent the different significance levels of the overrepresentation; yellow: FDR <0.05, orange: FDR <0.01, red: FDR <0.001.
(PDF)

S4 Fig Venn diagrams of differentially expressed genes in response to N-deficiency. A. Venn diagrams of N-deficiency stress-responsive genes in wild-type across four time points. B. Venn diagrams of N-deficiency stress-responsive genes in *rtcs* across four time points. C. Venn diagrams of N-deficiency stress-responsive genes in both genotypes.
(PDF)

S5 Fig Gene annotation of N-deficiency stress-responsive genes in both genotypes. Functional annotation for N-deficiency-responsive genes in both genotypes, and the top 50 GO terms according to the DEGs numbers were shown.
(PDF)

S6 Fig Correlation between RNA-Seq and qPCR data. Each RNA-Seq expression data was plotted against that from quantitative real-time PCR and fit into a linear regression. Both x- and y-axes were shown in log2 scale and each color represented a different gene.
(PDF)

S1 Table The detailed components of the nutrient solution. (XLSX)

S2 Table Primers of qRT-PCR assay used for differential expression genes in this study. (XLSX)

S3 Table Summary of sequencing data quality. (XLSX)

S4 Table Summary of read numbers and mapping rate based on the RNA-Seq data. Total reads: Total number of reads. Total mapped: Total number of reads mapped on the maize genome. Multiple mapped: Total number of reads mapped to multiple locations in maize genome. Uniquely mapped: Total number of reads mapped to uniquely locations in the maize genome. Read-1: The two directions of the paired-end sequencing. Read-1: The two directions of the paired-end sequencing. Reads map to '+': Total number of reads mapped to positive strand of maize genome. Reads map to '-': Total number of reads mapped to negative strand of maize genome.
(XLSX)

S5 Table Summary of all expression genes. (XLSX)

S6 Table Co-modulated differential expression genes between wild-type and *rtcs* under normal-N condition across four time points. (XLSX)

S7 Table GO annotation of co-modulated DEGs between wild-type and *rtcs* under normal-N condition across four time points. (XLSX)

S8 Table Co-modulated differential expression transcription factors between wild-type and *rtcs* under normal-N condition across four time points. (XLSX)

S9 Table Genotype-specific genes containing *LBD* motif. (XLSX)

S10 Table Differential expression genes in response to N deficiency. (XLSX)

S11 Table KEGG analysis of differential expression genes in maize roots responsive to N deficiency. (XLSX)

S12 Table DEGs associated with root development participate in response to N stress. (XLSX)
