# Supplementary figures and images for: Comparative RNA-Seq Analysis Reveals That Regulatory Network of Maize Root Development Controls the Expression of Genes in Response to N Stress
Source: PLoS One. 2016 Mar 18;11(3):e0151697. doi: 10.1371/journal.pone.0151697 (PMC4798287; doi:10.1371/journal.pone.0151697)

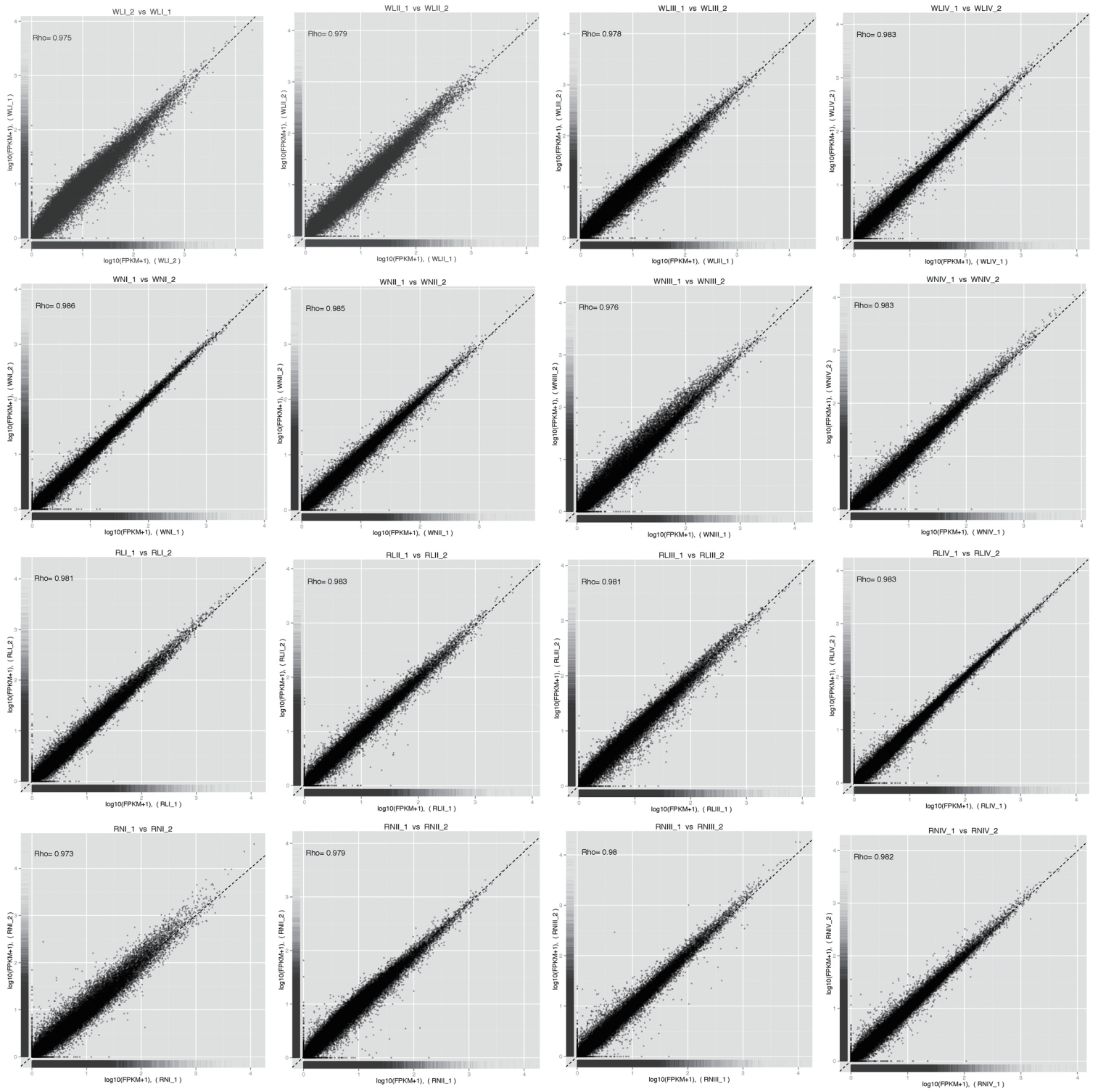

Supplement: S1 Fig — (PDF) [file pone.0151697.s001.pdf]

KEGG\_pathway

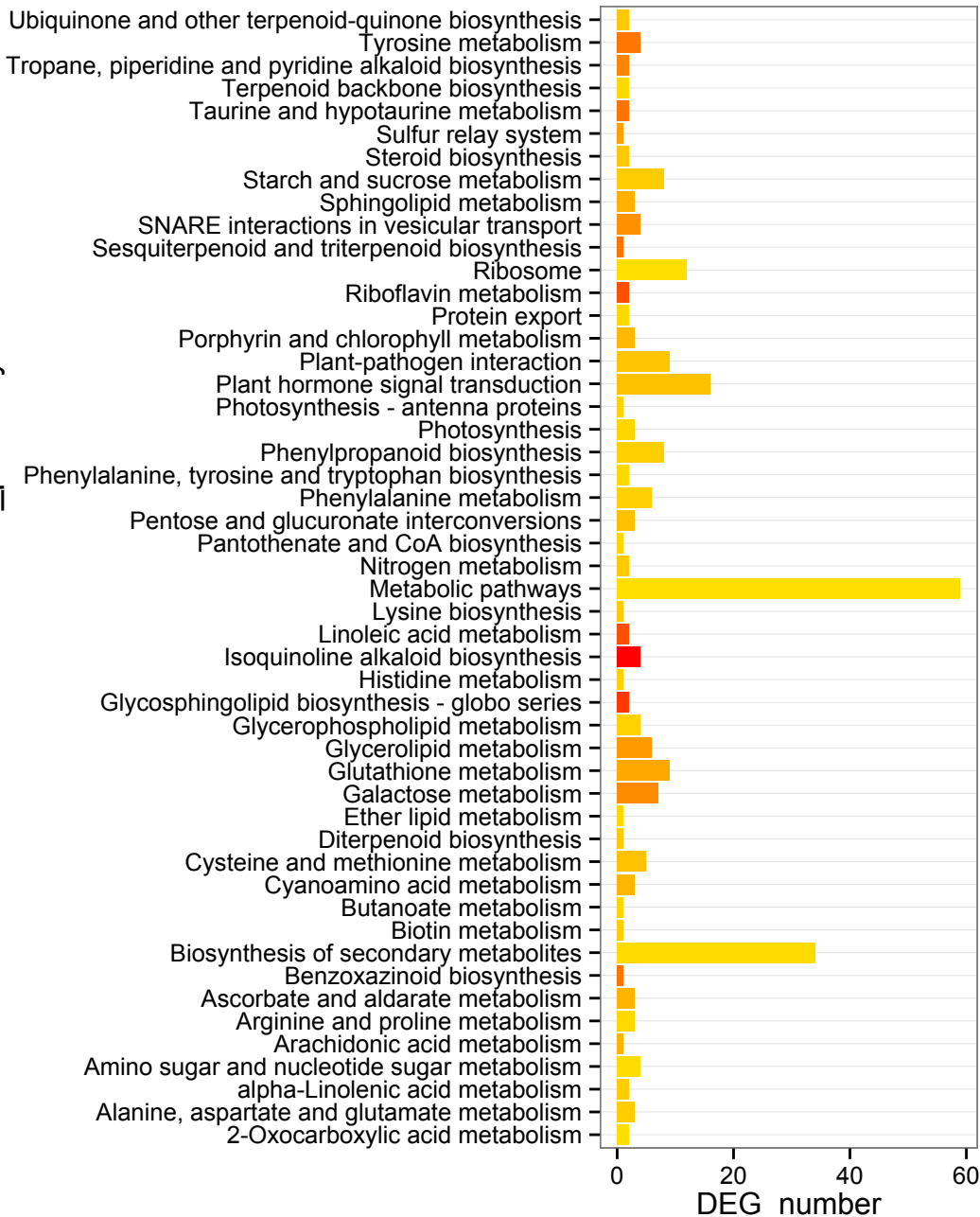

Rich\_factor

0.15

0.10

0.05

0

DEG\_number

60

Supplement: S2 Fig — The y-axis corresponds to KEGG Pathway, and the x-axis shows DEGs number. The color of the dot represent enrichment factor. (PDF) [file pone.0151697.s002.pdf]

## Biological processes

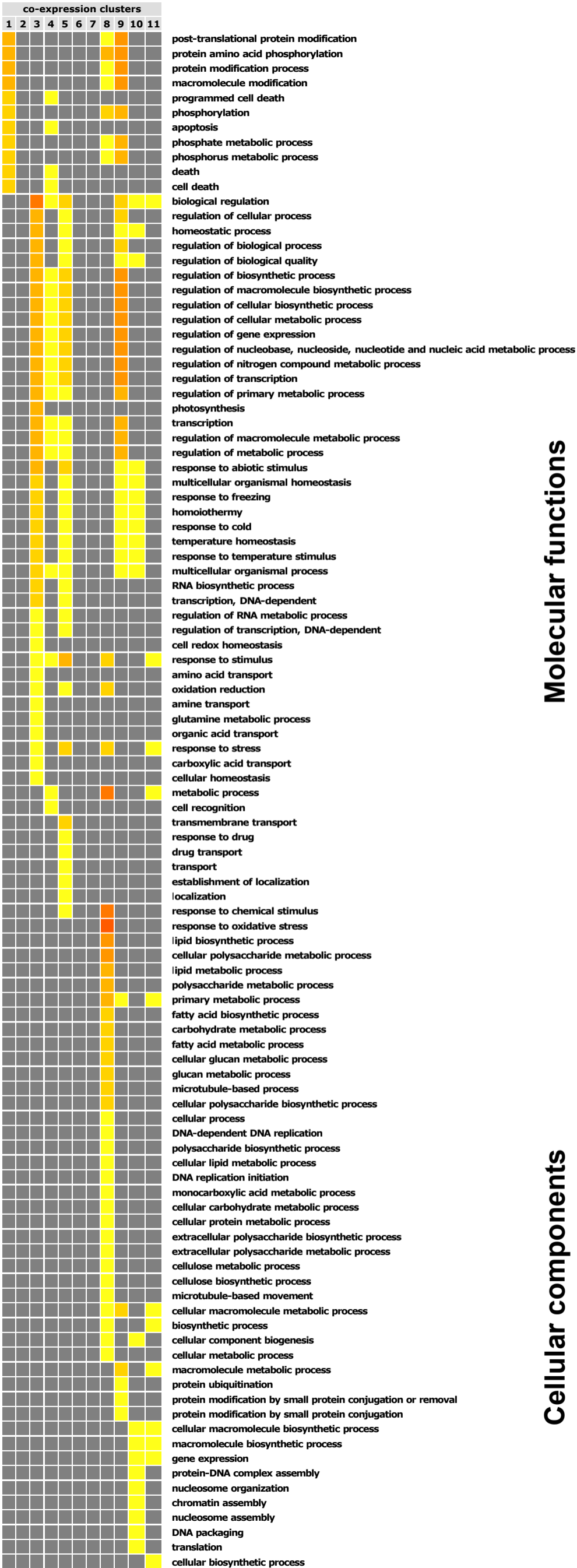

# Molecular functions

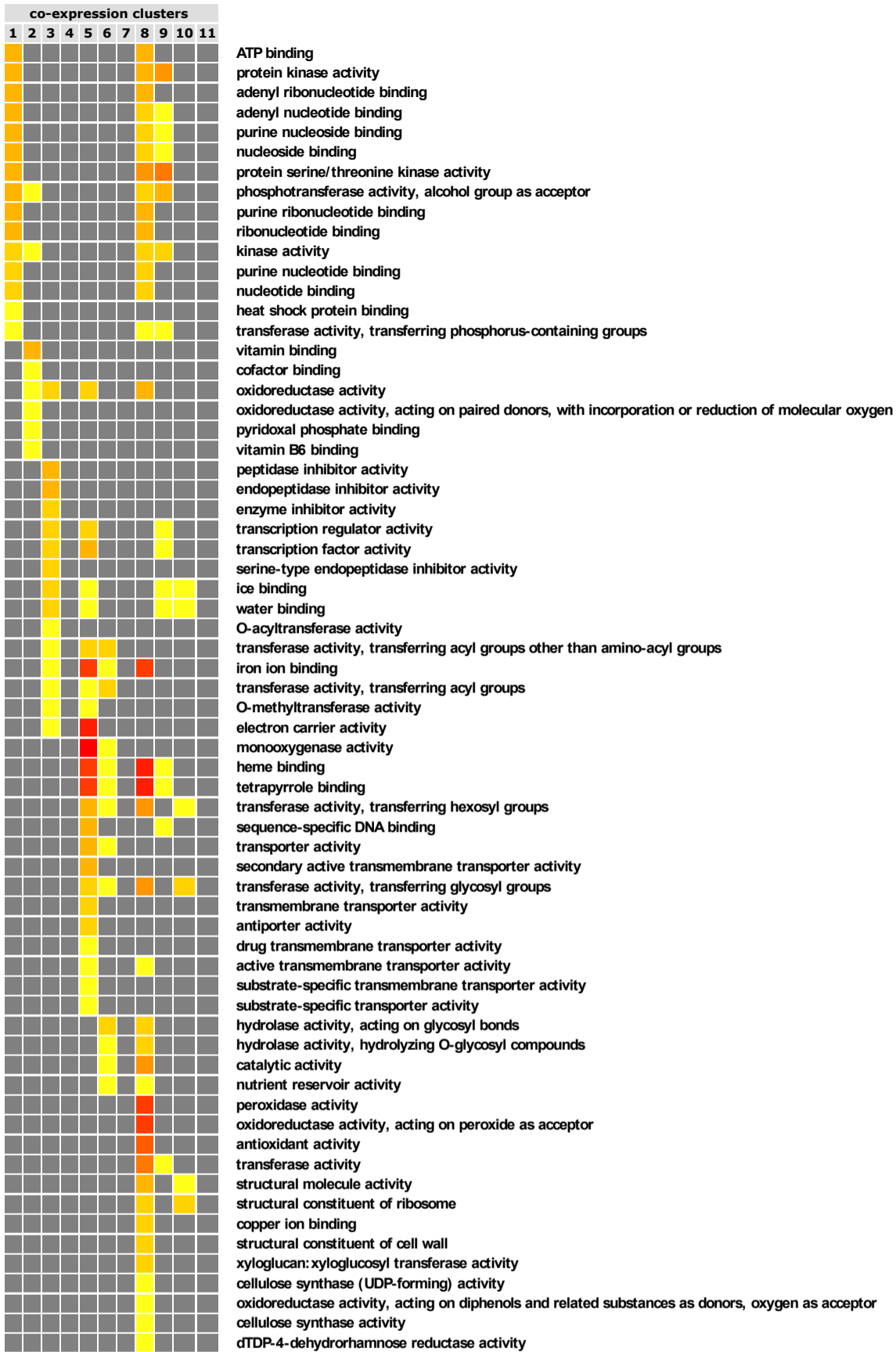

# Cellular components

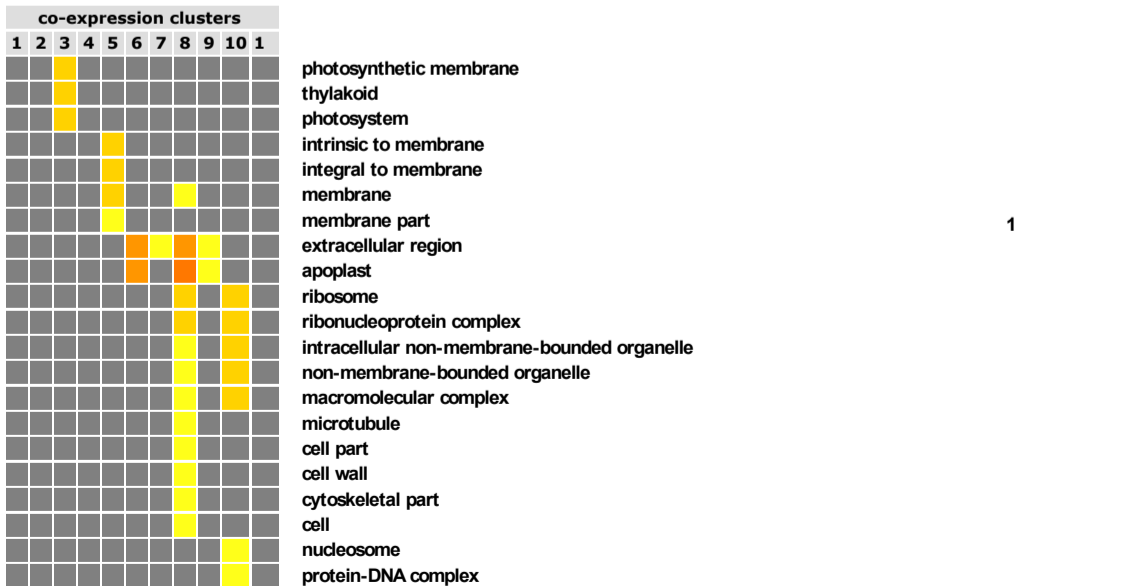

Supplement: S3 Fig — Different colors in the block represent the different significance levels of the overrepresentation; yellow: FDR <0.05, orange: FDR <0.01, red: FDR <0.001. (PDF) [file pone.0151697.s003.pdf]

**A**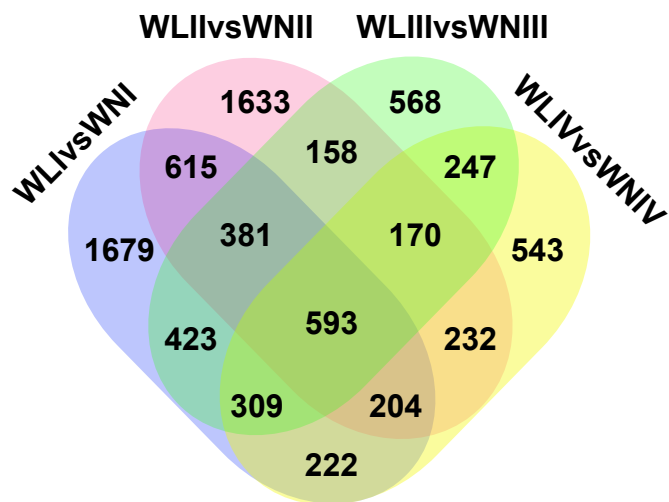**B**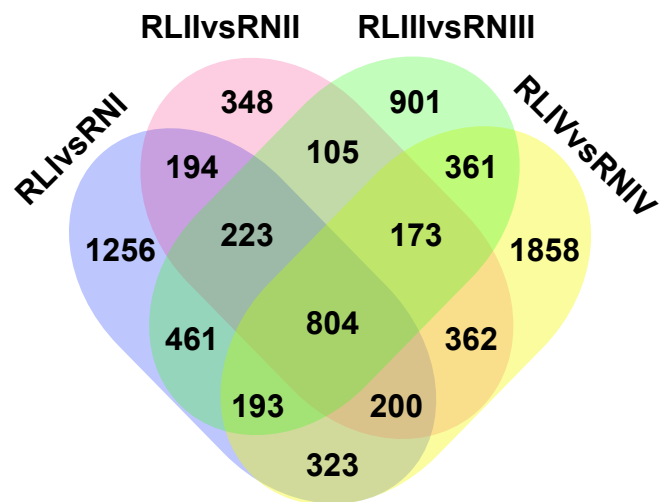**C**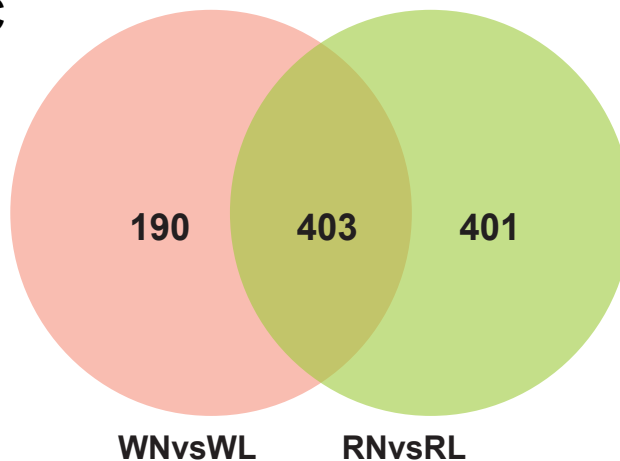

Supplement: S4 Fig — A. Venn diagrams of N-deficiency stress-responsive genes in wild-type across four time points. B. Venn diagrams of N-deficiency stress-responsive genes in rtcs across four time points. C. Venn diagrams of N-deficiency stress-responsive genes in both genotypes. (PDF) [file pone.0151697.s004.pdf]

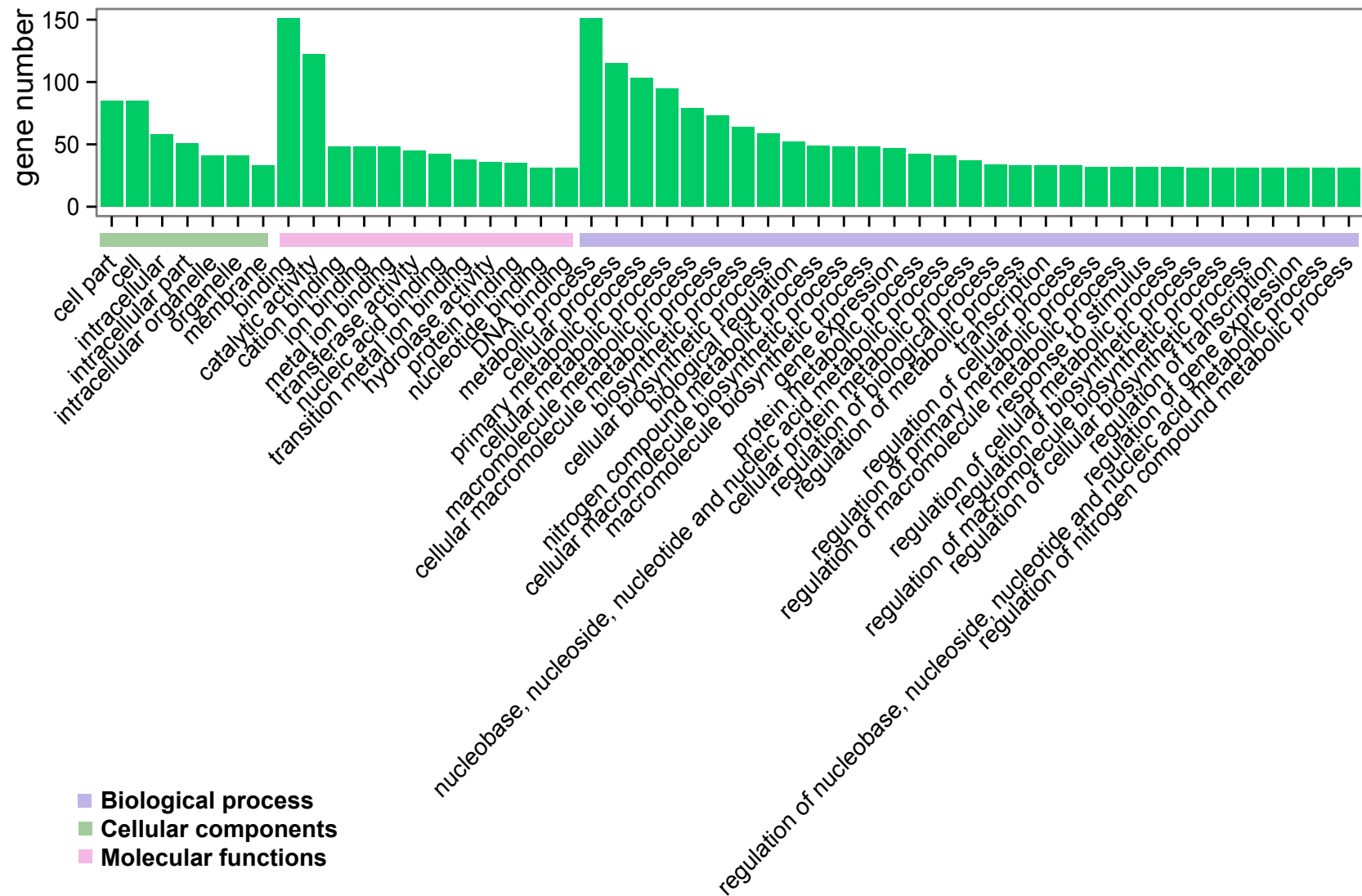

Supplement: S5 Fig — Functional annotation for N-deficiency-responsive genes in both genotypes, and the top 50 GO terms according to the DEGs numbers were shown. (PDF) [file pone.0151697.s005.pdf]

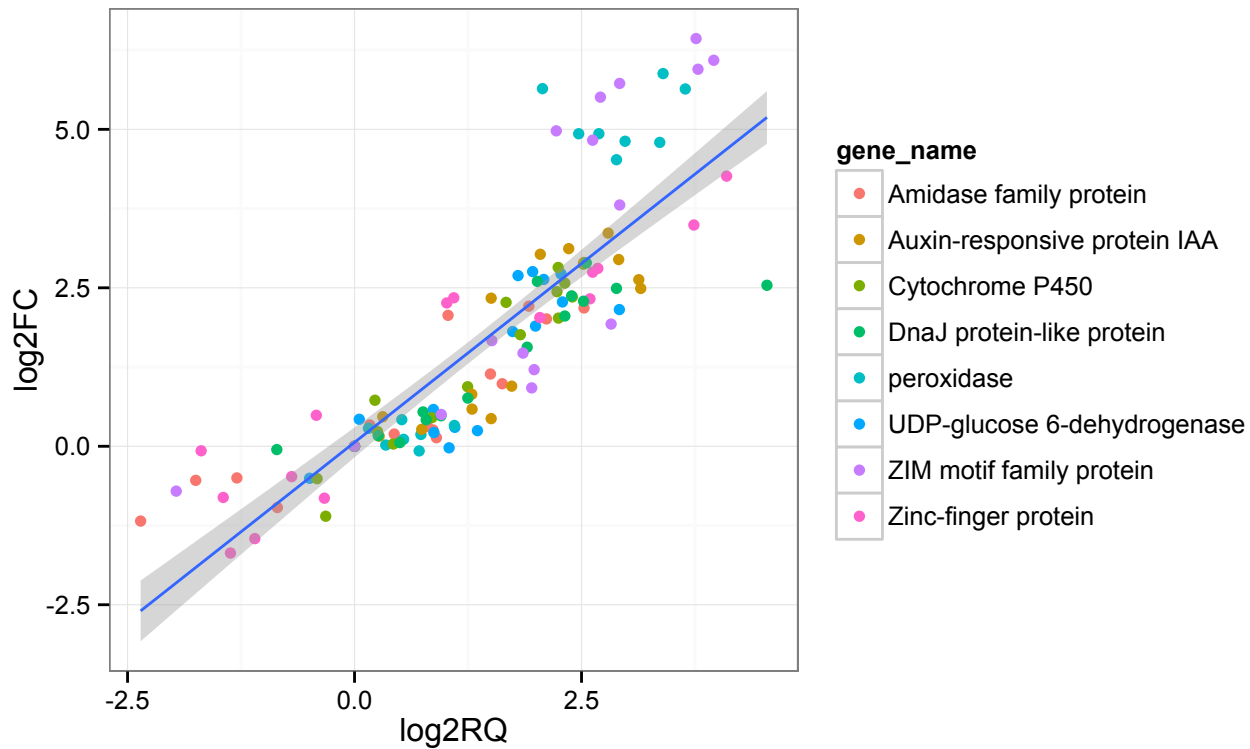

Supplement: S6 Fig — Each RNA-Seq expression data was plotted against that from quantitative real-time PCR and fit into a linear regression. Both x- and y-axes were shown in log2 scale and each color represented a different gene. (PDF) [file pone.0151697.s006.pdf]
